# Prevention of Disease

**Published:** 1884-06

**Authors:** 


### PREVENTION OF DISEASE.

THERE has been much time and labor wasted in endeavoring to teach men how to avoid disease. As a rule, it is only the "burnt child that dreads the fire." We constantly meet persons who are the mere wrecks of their former selves, through imprudences and diseases that were avoidable. Does anyone suppose that these serve as admonitions that many others will profit by? Have not the sufferers themselves had timely warnings without number of the inevitable results of wrong living? As "men think all men mortal but themselves," so in their strength they seem to think, as a rule, that evil will come to others sooner than to themselves. It is this self-confidence, inspired by years of uninterrupted health, that often induces recklessness. We sometimes look with admiration at the working of a machine; perhaps a steam engine. It seems like a thing of life. How quickly it responds to the various movements of the valves, worked by the skillful engineer: with what precision does it run; not a jar or a creak is to be heard. Soon, what we at first watched with so much interest seems monotonous, and even tiresome;—just as the most faultless weather becomes almost unendurable if it continues for weeks and months without interruption. Whether it be a steam engine, or that more wonderful mechanism the human frame, that depends for safety on our watchfulness, an incautious move, a slight neglect of duty, and it soon becomes a wreck. Extremists are responsible for half the mischief that is wrought among those who strive to maintain health by proper living. There are some who in particular prove their faith in good living by eating and drinking too much, habitually; a much larger class of so-called reformers starve body and mind by efforts to live without substantial nourishment. Some one has invented a method of utilizing the waste from coal mines by mixing it with clay. Such fuel gives out a moderate heat, and answers certain purposes; but no one seems to have succeeded in burning clay *without* the coal dust. And thus it is in nourishing the body. Man can not live on crackers and water, and be of any use to himself, or service to others; but let him mix the clay and the coal—that is, let him live on a mixed diet and avoid all excesses, and he will attain the full measure of health and usefulness, so far as foods are concerned. But there is something else a person must have a constant supply of or health will not remain long with him; that is pure, fresh air. No one should remain an hour in any room where there is not an ample and unobstructed avenue to the air outside. There is still another element that is indispensable to health—water. Pure water is the best and most wholesome of all solvents. A copious draft of cold water is the best of laxatives; and when taken half an hour before or after meals, is a promoter of digestion. Its value as a *detergent* has been recognized from the earliest ages. The bath marked a high civilization; and although "cleanliness is next to Godliness" is not a scriptural quotation, it was the sentiment of one of the greatest christian reformers.

A single word gives expression to a golden rule. That word is—MODERATION. It is of more general application than a volume of rules of life with that word left out. It is applicable to food, dress, sleep, exercise, and to all the affairs of life. There is one other rule, not inconsistent with it—"When you don't know what to do, Do NOTHING." When you are ill do nothing until you ascertain the nature of the illness, and can be treated by a competent physician. There is greater safety in rest—in doing nothing for a time—than in hap-hazard treatment. It is easier and safer to do nothing until you know what to do, than to rectify mistakes.

